# Supplementary material for: Protease-controlled secretion and display of intercellular signals
Source: Nat Commun. 2022 Feb 17;13:912. doi: 10.1038/s41467-022-28623-y (PMC8854555; doi:10.1038/s41467-022-28623-y)
Supplement: Supplementary file 1 — Supplementary Information [file 41467_2022_28623_MOESM1_ESM.docx]

**Supplementary Information:** Protease-controlled secretion and display of intercellular signals

Alexander E. Vlahos^1^, Jeewoo Kang^2^, Carlos A. Aldrete^1^, Ronghui Zhu^3^, Lucy S. Chong^3^, Michael B. Elowitz^3^, and Xiaojing J. Gao^1,2^*

1) Department of Chemical Engineering, Stanford University, Stanford, CA, 94305

2) Neurosciences Interdepartmental Program, Stanford University, Stanford, CA, 94305

3) Howard Hughes Medical Institute, Division of Biology and Biological Engineering, California Institute of Technology, Pasadena, CA 91125,

*Corresponding author: [xjgao@stanford.edu](mailto:xjgao@stanford.edu)

**Supplementary Figures:**


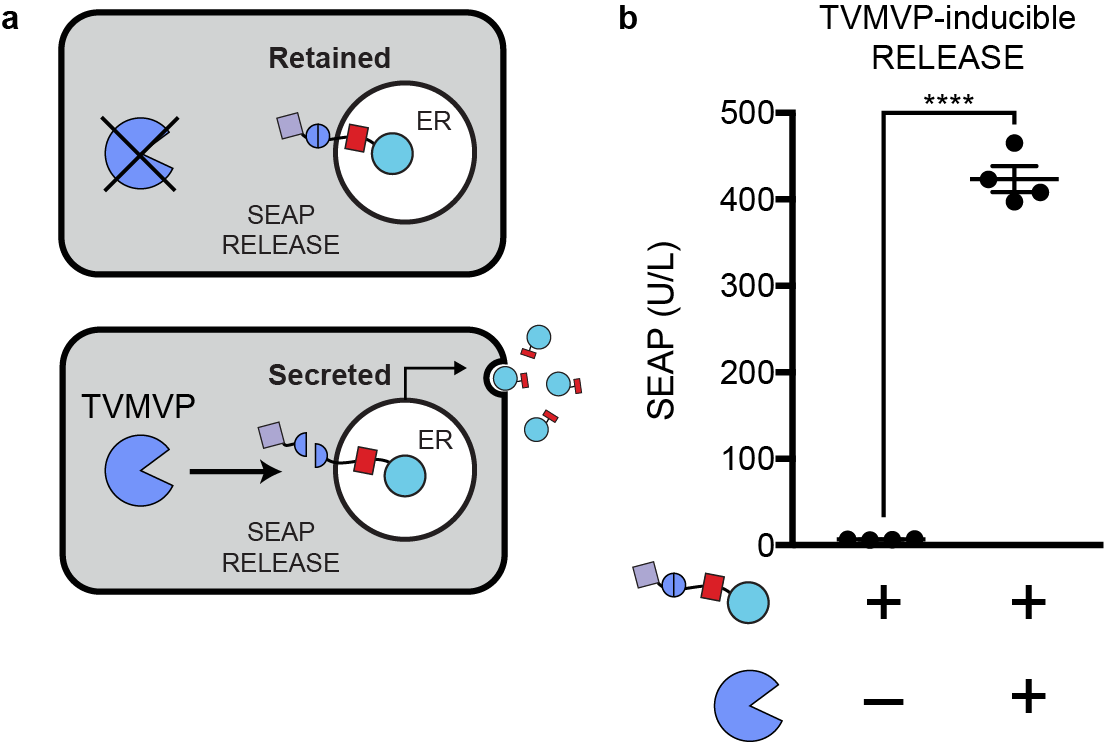


**Supplementary Figure 1: TVMVP-inducible RELEASE.** **a)** Schematic of TVMVP-inducible RELEASE for controlling protein secretion. **b)** SEAP was fused to a TVMVP-inducible RELEASE, and co-expression with TVMVP secreted more SEAP. Each dot represents a biological replicate. Mean values were calculated from four biological replicates (**b**) +/- SEM. The results are representative of at least two independent experiments; significance was tested using an unpaired two-tailed Student’s *t*-test between the two indicated conditions. **** = p < 0.0001.


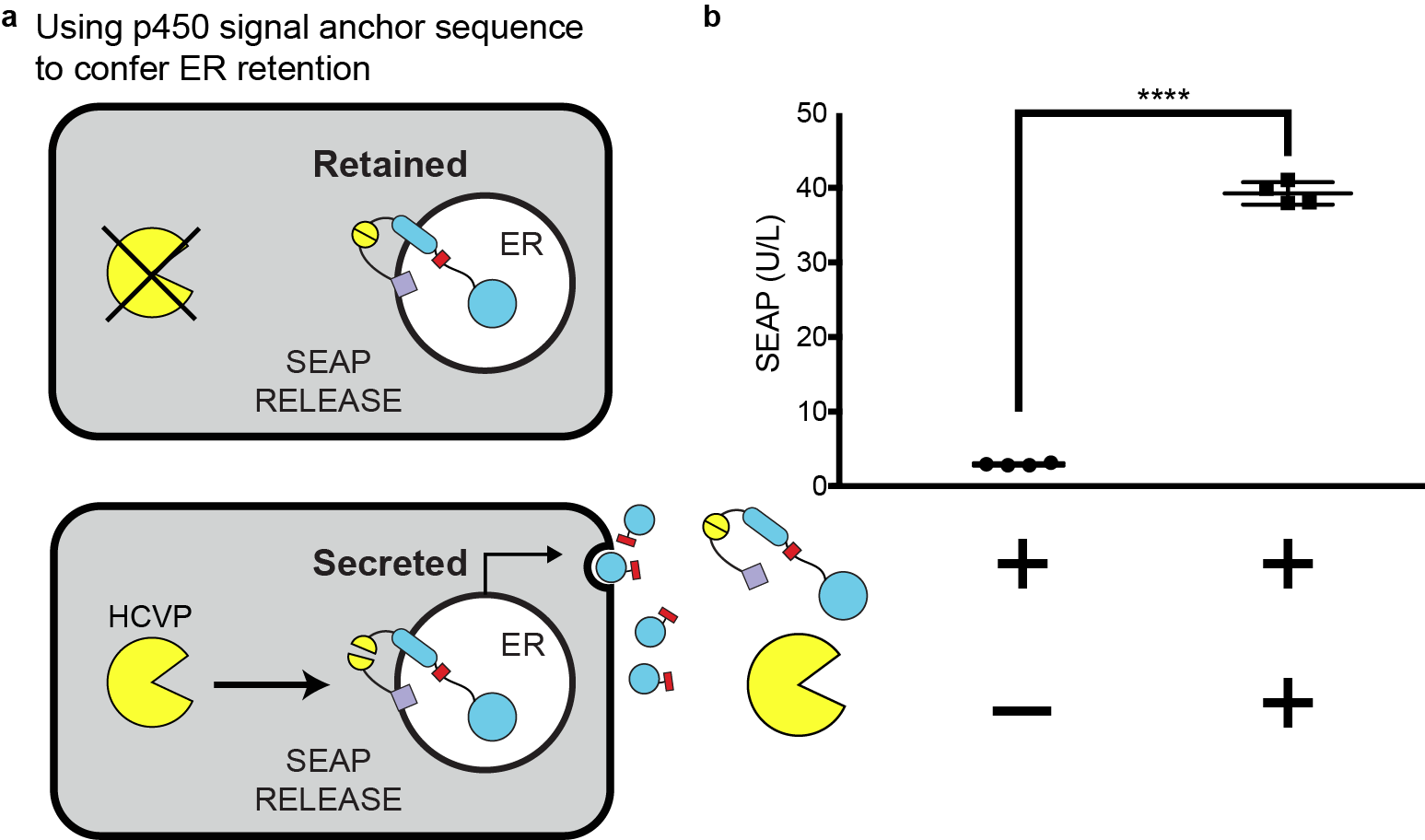


**Supplementary Figure 2:** An alternative ER-retention domain to create RELEASE. **a)** Schematic of RELEASE using the N-terminal signal anchor sequence of cytochrome p450 to control protein secretion. **b)** When co-expressed with HCVP, SEAP secretion increased relative to when the HCVP was absent. Each dot represents a biological replicate. Mean values were calculated from four biological replicates (**b**) +/- SEM. The results are representative of at least two independent experiments; significance was tested using an unpaired two-tailed Student’s *t*-test between the two indicated conditions for each experiment. **** = p < 0.0001.


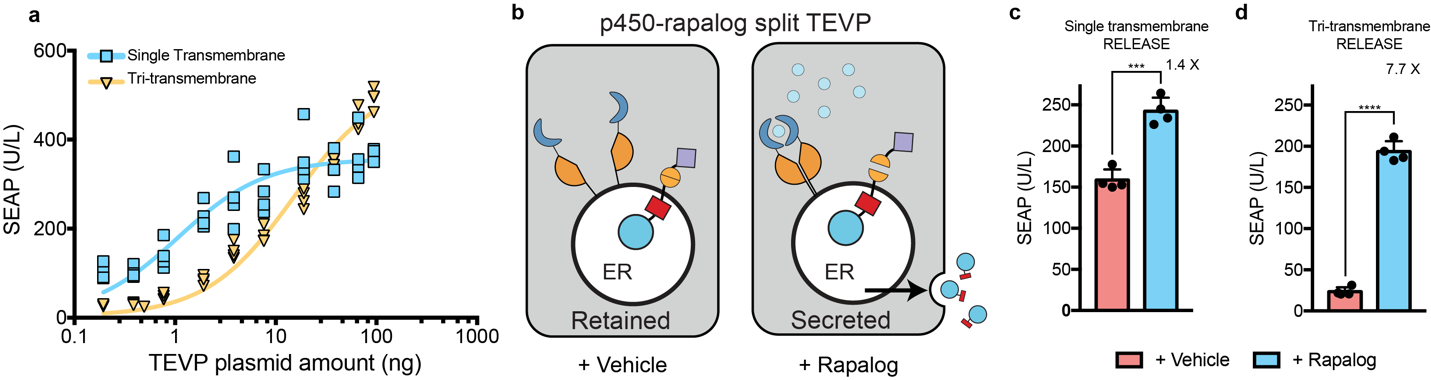


**Supplementary Figure 3:** Dynamic range of RELEASE is increased by using different RELEASE constructs that have **a)** different cleavage efficiencies. **b)** Schematic of rapalog-inducible split TEVP localized to the ER membrane via the p450 signal anchor sequence. When rapalog is present, split TEVP will be reconstituted to cleave SEAP RELEASE. **c)** With the single transmembrane RELEASE construct, there was a minor increase in the SEAP secretion after induction with rapalog relative to the control. **d)** Using the tri-transmembrane RELEASE construct there was a greater difference in SEAP secretion compared to the single transmembrane RELEASE construct (7.7-fold vs. 1.4-fold). The difference between the fold-changes was attributed to the reduction in SEAP secretion under basal conditions with the tri-transmembrane RELEASE. Each dot represents a biological replicate. Mean values were calculated from four biological replicates (**c, d**) +/- SEM. The results are representative of at least two independent experiments; significance was tested using an unpaired two-tailed Student’s *t*-test between the two indicated conditions for each experiment. *** = p < 0.001, **** = p < 0.0001


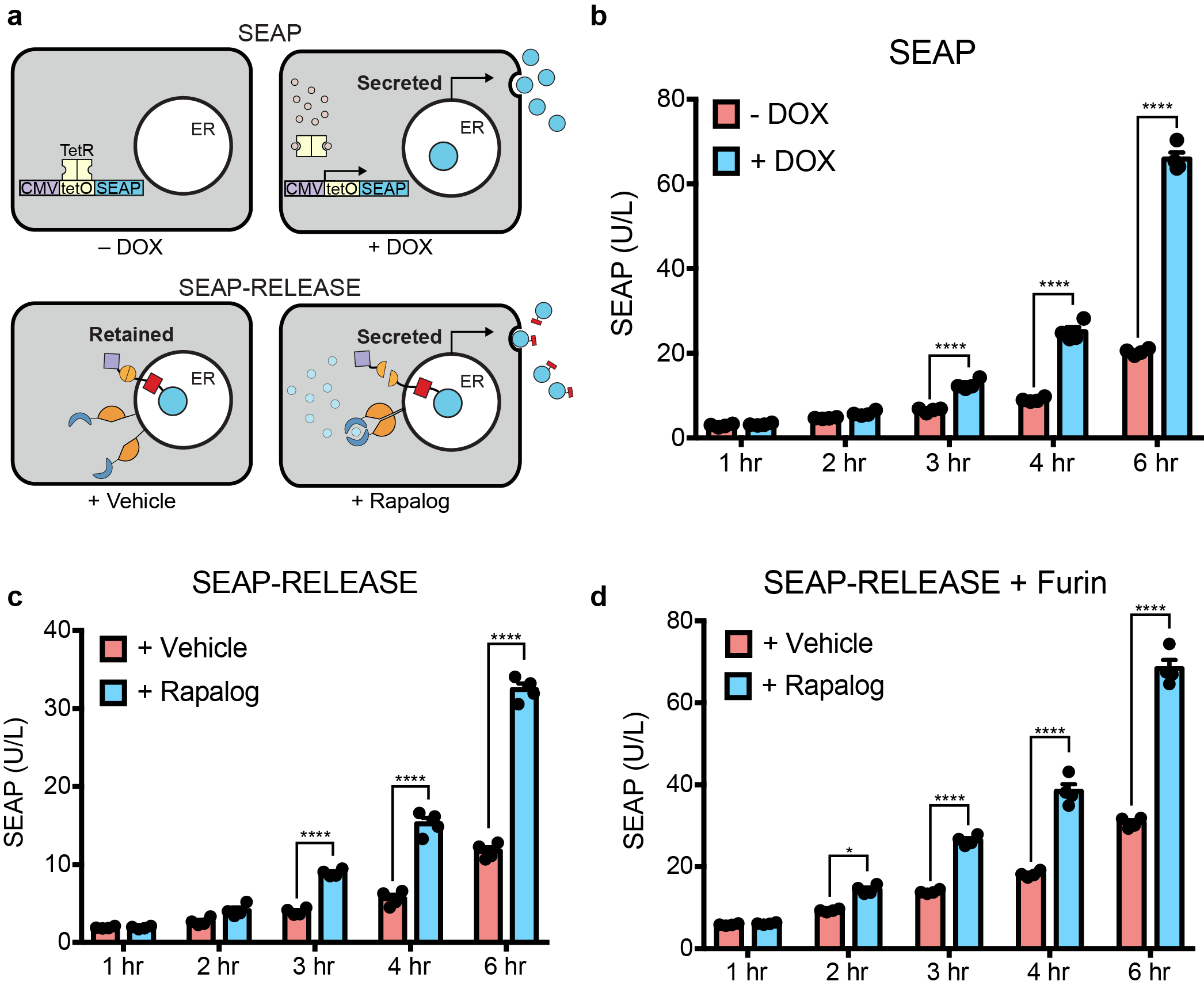


**Supplementary Figure 4:** **Dynamics of protein secretion using traditional inducible gene expression systems and RELEASE.** a) Schematic of cells transfected with DOX-inducible SEAP, and SEAP-RELEASE and Rapalog-inducible split-TEVP. 48 hours after transfection, cells were induced with DOX or Rapalog, and the supernatant was collected. **b)** A significant difference in SEAP secretion was first observed 3 hours post-induction between cells induced with DOX and the uninduced controls. **c)** Using SEAP-RELEASE, a significant difference between induced and uninduced cells was observed beginning at 3 hours post-induction with rapalog. **d)** Through overexpression of the furin endoprotease, increased SEAP secretion was observed as quickly as 2 hours after induction using Rapalog compared to cells induced with the vehicle. The vehicle for rapalog was 95% EtOH. Each dot represents a biological replicate. Mean values were calculated from four biological replicates (**b, c, d**) +/- SEM. The results are representative of at least two independent experiments; significance was tested using by two-way ANOVA with a Tukey’s post-hoc comparison test among the multiple conditions. * = p < 0.05, **** = p < 0.0001.


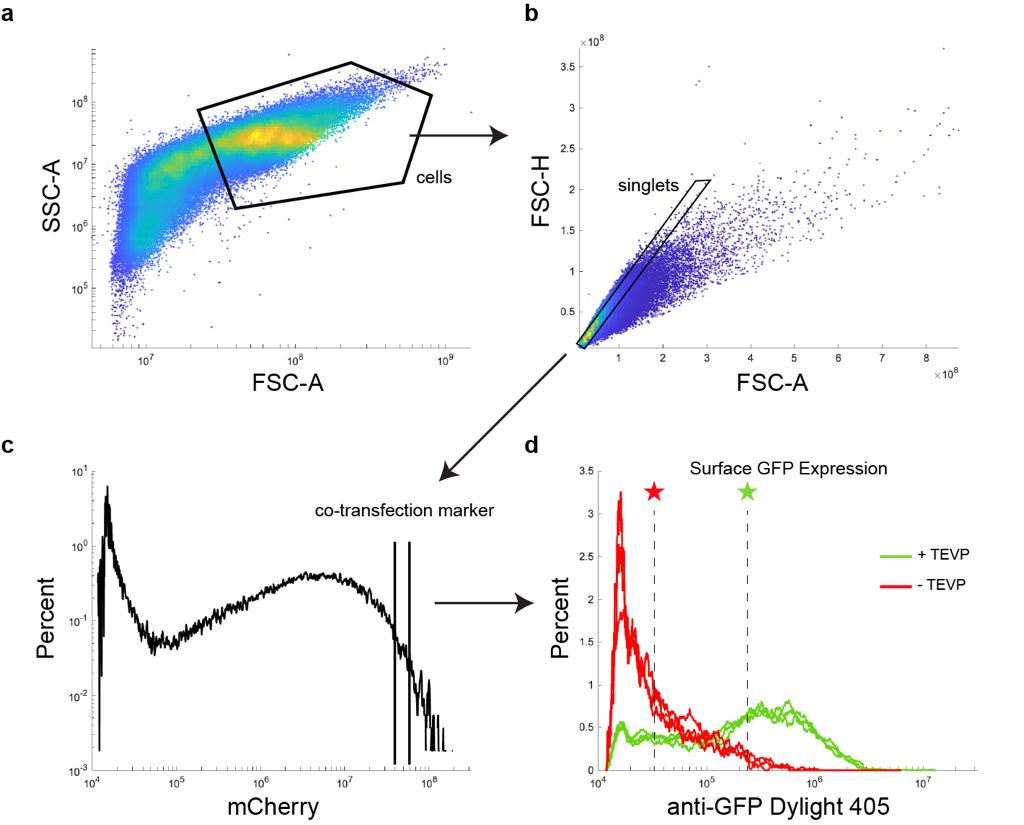


**Supplementary Figure 5: Gating strategy for flow cytometry analysis.** **a)** Cells were gated based on FSC-A and SSC-A, followed by gating for singlets **b)**. Each experiment uses a co-transfection marker, such as mCherry to select for highly transfected cells (~5% of the cellular population) for analysis. **d**) Representative experiment analyzing the amount of surface GFP expression using RELEASE with and without TEVP (data for **Fig. 1h – left panel**). The green and red stars represent the median fluorescence intensities of cells containing GFP fused to RELEASE with and without TEVP, respectively.


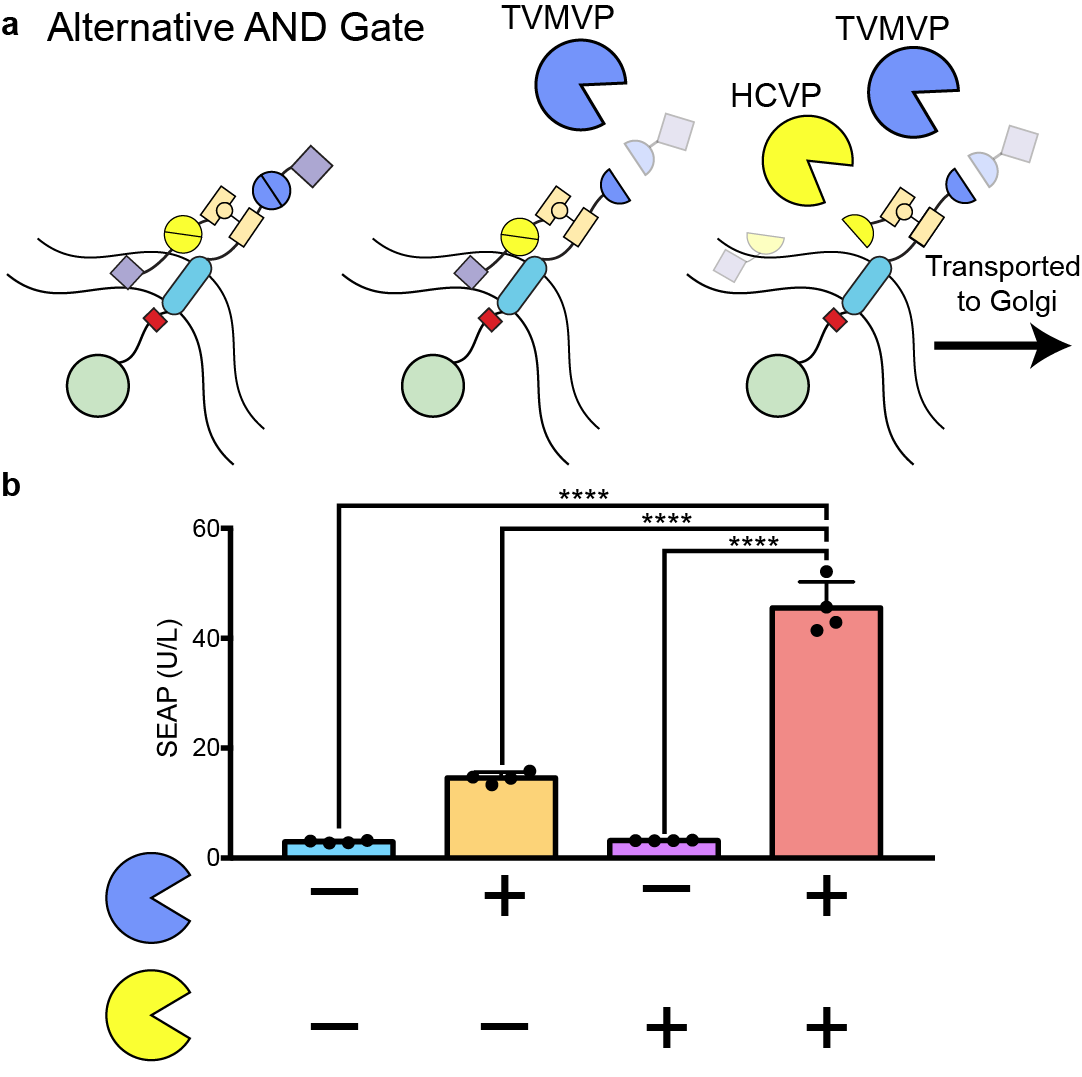


**Supplementary Figure 6:** An alternative AND gate was implemented using the SpyTag/SpyCatcher peptide-protein pair. HCVP inducible SpyCatcher was localized to the ER using the signal anchor sequence of p450. A TVMVP inducible RELEASE construct containing an internal SpyTag peptide within the cytoplasmic linker region rapidly associated with the ER-retained SpyCatcher. SEAP secretion was dependent on the expression of both HCVP and TVMVP, however some SEAP was secreted when co-expressing TVMVP alone, which may be due to an incomplete reaction with SpyCatcher. Each dot represents a biological replicate. Mean values were calculated from four biological replicates (**b**) +/- SEM. The results are representative of at least two independent experiments; significance was tested by one-way ANOVA with a Tukey’s post-hoc comparison test among the multiple conditions. **** = p < 0.0001.


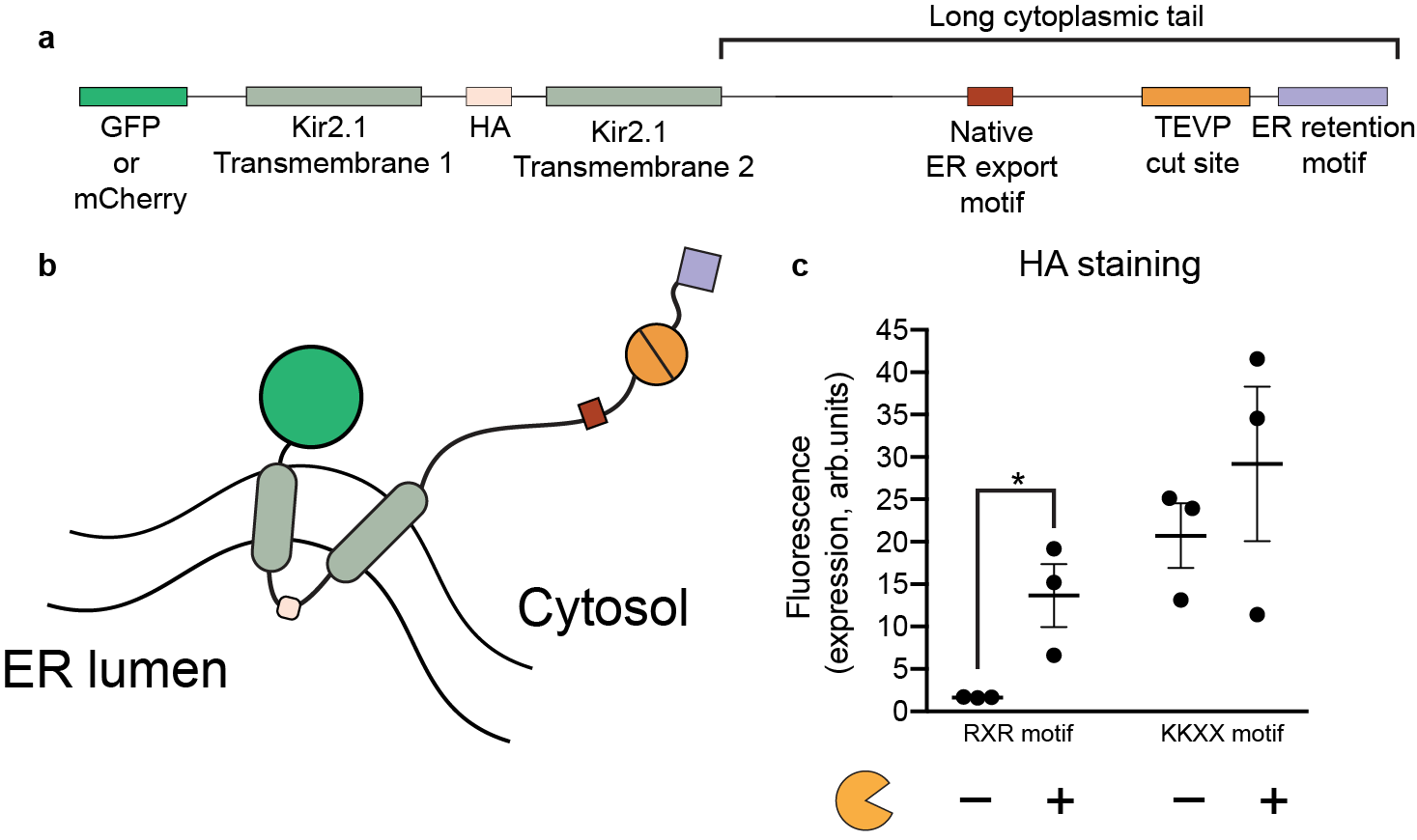


**Supplementary Figure 7:** Surface display of Kir2.1 was dependent on the ER retention motif used in the RELEASE construct. **a**) Schematic of Kir2.1 RELEASE. The hemagglutinin (HA) epitope was incorporated into the extracellular loop to measure the surface expression of Kir2.1 using flow cytometry. **b**) Due to the large cytoplasmic tail of Kir2.1, the C-terminus was farther away from the ER membrane relative to other RELEASE constructs. **c**) The RXR motif retains proteins better than the KKXX motif when the C-terminal is distal to ER membrane. Each dot represents a biological replicate. Mean values were calculated from three biological replicates (**c**) +/- SEM. The results are representative of at least two independent experiments; significance was tested using an unpaired two-tailed Student’s *t*-test between the two indicated conditions for each experiment. * = p < 0.05.


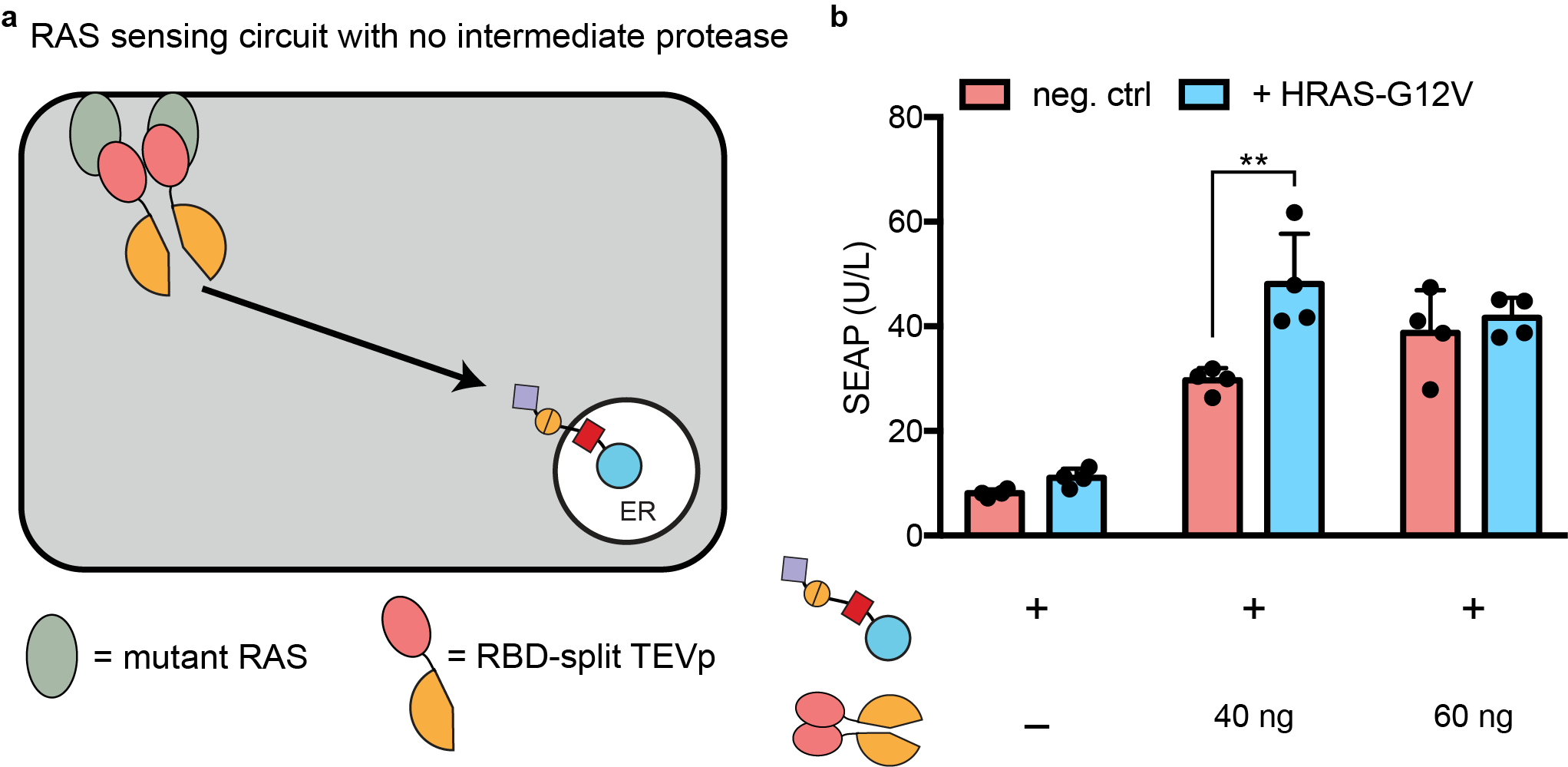


**Supplementary Figure 8:** **a)** Schematic of RAS-sensing circuit without using intermediate protease to propagate the signal. **b)** The sensing of active mutant HRAS-G12V at the membrane using RBD-split TEVP showed a significant increase in SEAP secretion when transfecting 40 ng of the sensor, relative to wildtype HEK293 cells. This change in secretion was observed when transfecting 60 ng of the RBD-split TEVP sensor. Each dot represents a biological replicate. Mean values were calculated from four biological replicates (**b**) +/- SEM. The results are representative of at least two independent experiments; significance was tested by two-way ANOVA with a Tukey’s post-hoc comparison test among the multiple conditions. ** = p < 0.01.


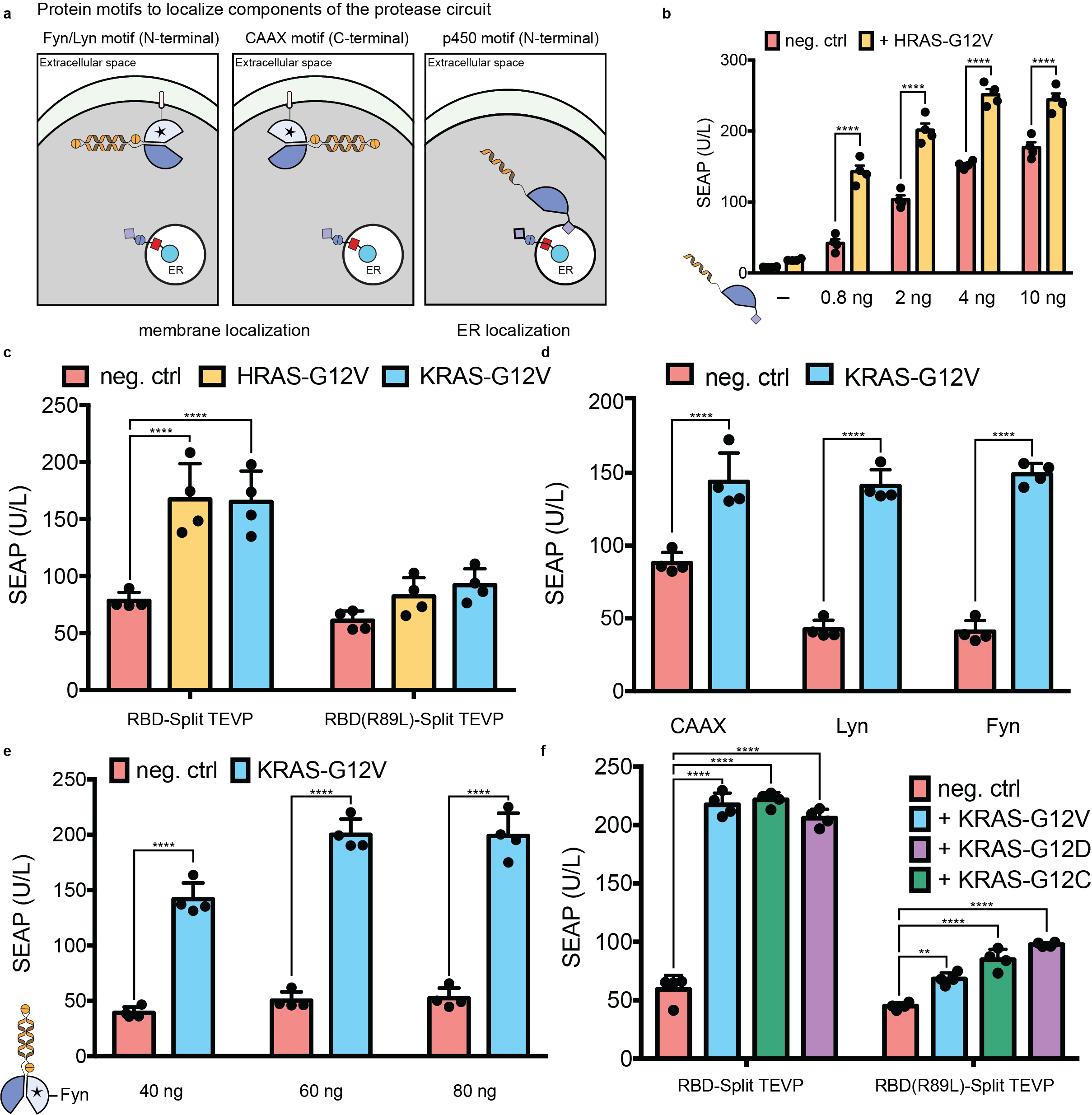


**Supplementary Figure 9:** **a)** To efficiently propagate information from the cell membrane to the ER, signalling motifs were incorporated to localize components of the intermediate protease to the membrane via the Fyn/Lyn motif (left panel), or CAAX motif (middle panel). In addition, we used the signal anchor sequence of cytochrome p450 to localize components to the ER membrane (right panel). **b)** To increase the dynamic range of the RAS-sensing circuit (topology 3 from **Fig. 4d**), the amount of the ER-localized split TVMVP was reduced. **c)** The RAS-sensing circuit was comparable for sensing other RAS isoforms, such as KRAS-G12V. **d)** The Fyn and Lyn membrane associating motifs had reduced background relative to the CAAX motif, and **e)** increasing the amount of the membrane-associated TVMVP half localized with the Fyn motif, improved the dynamic range. **f)** The RAS-sensing circuit sensed other active mutants of KRAS at comparable levels to the KRAS-G12V mutant. Each dot represents a biological replicate. Mean values were calculated from four biological replicates (**b-f**) +/- SEM. The results are representative of at least two independent experiments; significance was tested by two-way ANOVA with a Tukey’s post-hoc comparison test among the multiple conditions. ** = p < 0.01, **** = p < 0.0001.


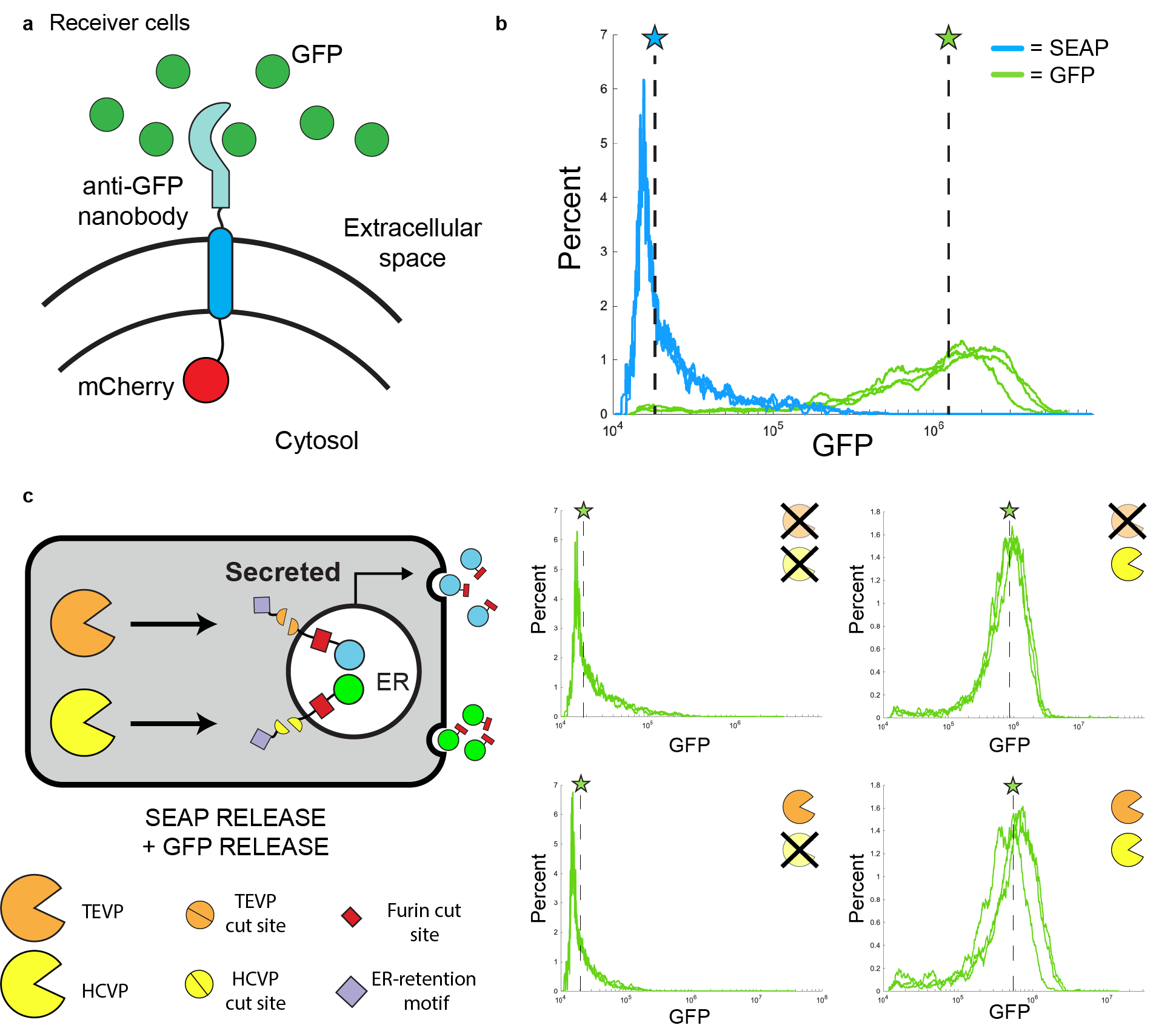


**Supplementary Figure 10:** **a)** Schematic of cells transfected with the Gbp6 anti-GFP nanobody conjugated to mCherry. **b)** Receiver cells were incubated with cell free supernatant from various RELEASE conditions to validate that they could capture GFP. Supernatants from cells that constitutively secreted SEAP or GFP alone, were used as negative and positive controls, respectively. **c)** Raw flow plots from **Fig. 2b**, quantifying the amount of captured GFP on the receiver cells that were incubated with supernatant from cells co-expressed with SEAP-RELEASE, GFP-RELEASE, and different combinations of the cognate proteases. The stars represent the median fluorescence intensities for each group.


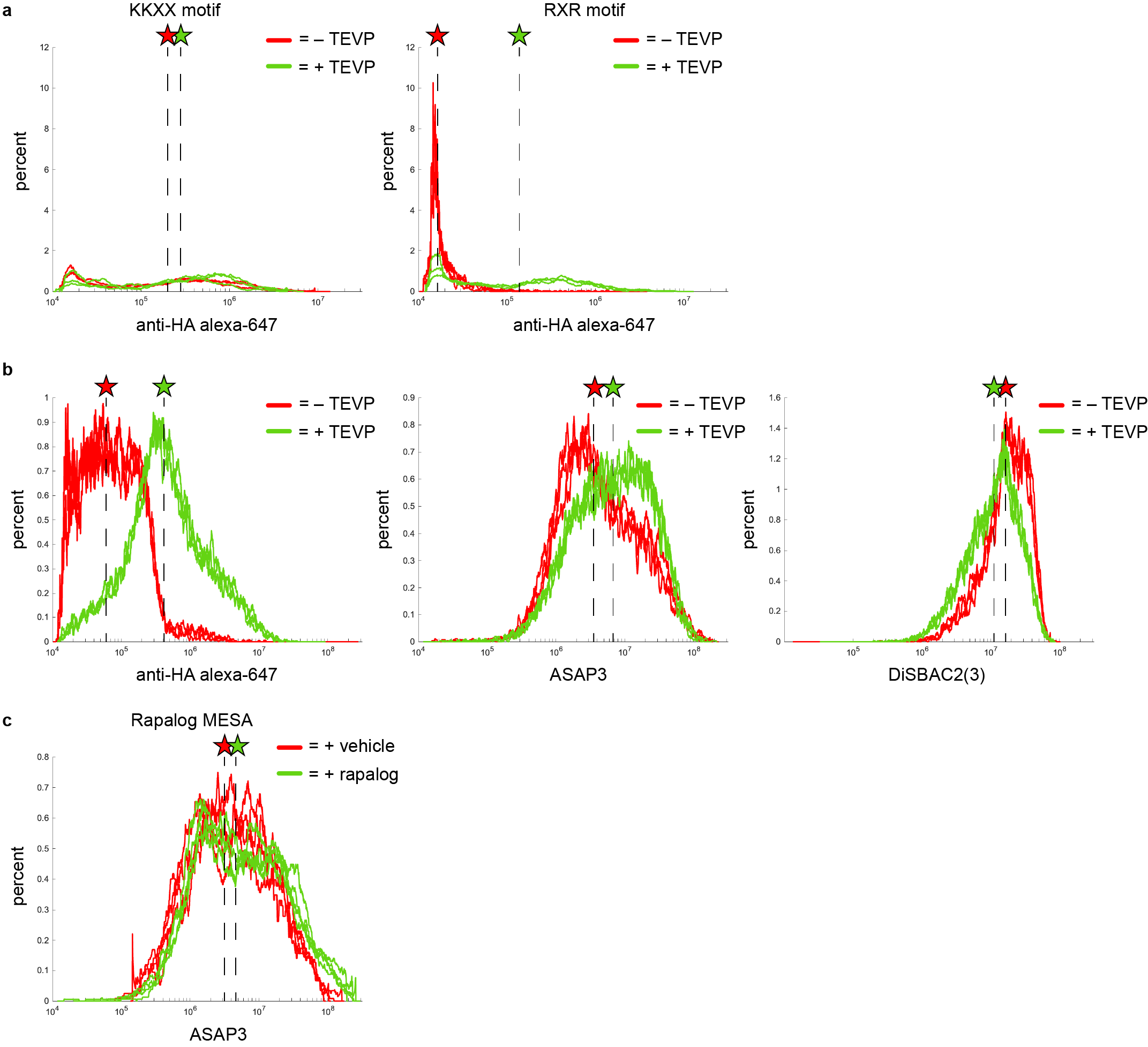


**Supplementary Figure 11:** Raw flow plots from **a)** **supplementary fig. 7c**, quantifying the amount of Kir2.1 present on the surface using an anti-HA antibody conjugated to alexa-647. Raw flow plots from **b)** **fig. 3d, e, f**, quantifying the amount of Kir2.1 present on the surface and changes in membrane potential, respectively. Raw flow plots from **c)** **fig. 5e** quantifying changes in membrane potential with cells expressing rapalog MESA receptor and ASAP3. The stars represent the median fluorescence intensities for each group.

**Supplementary Tables:**

| **RELEASE Plasmid** | **Protease Cut Site** | **Apparent Cleavage Efficiency (amount (ng) of plasmid to achieve ½ Vmax) +/- S.E.** |
| --- | --- | --- |
| CMVTO-SEAP-26Sfur-3TM-tevs-KKMP | TEVP | 15.8 ± 1.6 |
| CMVTO-SEAP-26Sfur-B2AD-tevs-KKMP | TEVP | 1.1 ± 0.2 |
| CMVTO-SEAP-26Sfur-3TM-hcvs-KKMP | HCVP | 0.6 ± 0.1 |
| CMVTO-SEAP-26Sfur-B2AD-hcvs-KKMP | HCVP | 1.7 ± 0.2 |
| CMVTO-SEAP-26Sfur-3TM-hcvs-KKMP (GS) | HCVP | 5.5 ± 0.7 |
| CMVTO-SEAP-26Sfur-B2AD-hcvs-KKMP (GS) | HCVP | 113.9 ± 25.2 |
| CMVTO-SEAP-26Sfur-B2AD-tvmvs-KKMP | TVMVP | 7.7 ± 0.9 |

**Supplementary Table 1: Apparent cleavage efficiencies of different RELEASE constructs used in this study.** The apparently cleavage efficiencies of different RELEASE constructs were calculated by performing non-linear regression using the Michaelis-Menten equation. The cleavage efficiencies were represented by the K_m_ calculated from the fitted line. All non-linear regression was calculated using Prism 7.0.

**Supplementary Data:**

**Supplementary Data 1:** Experimental details.

**Supplementary Data 2:** List of plasmids and the amounts used in this study.

Please see attached excel file.
