# Supplementary material for: Protease-controlled secretion and display of intercellular signals
Source: Nat Commun. 2022 Feb 17;13:912. doi: 10.1038/s41467-022-28623-y (PMC8854555; doi:10.1038/s41467-022-28623-y)
Supplement: Supplementary file 2 — Reporting Summary [file 41467_2022_28623_MOESM2_ESM.pdf]

nature research

Corresponding author(s): Xiaojing Gao

Last updated by author(s): Jan 30, 2022

Reporting Summary

Nature Research wishes to improve the reproducibility of the work that we publish. This form provides structure for consistency and transparency in reporting. For further information on Nature Research policies, see our Editorial Policies and the Editorial Policy Checklist.

Statistics

For all statistical analyses, confirm that the following items are present in the figure legend, table legend, main text, or Methods section.

n/a	Confirmed	
☐	☒	The exact sample size (*n*) for each experimental group/condition, given as a discrete number and unit of measurement
☐	☒	A statement on whether measurements were taken from distinct samples or whether the same sample was measured repeatedly
☐	☒	The statistical test(s) used AND whether they are one- or two-sided<br>*Only common tests should be described solely by name; describe more complex techniques in the Methods section.*
☒	☐	A description of all covariates tested
☒	☐	A description of any assumptions or corrections, such as tests of normality and adjustment for multiple comparisons
☐	☒	A full description of the statistical parameters including central tendency (e.g. means) or other basic estimates (e.g. regression coefficient) AND variation (e.g. standard deviation) or associated estimates of uncertainty (e.g. confidence intervals)
☐	☒	For null hypothesis testing, the test statistic (e.g. *F*, *t*, *r*) with confidence intervals, effect sizes, degrees of freedom and *P* value noted<br>*Give P values as exact values whenever suitable.*
☒	☐	For Bayesian analysis, information on the choice of priors and Markov chain Monte Carlo settings
☒	☐	For hierarchical and complex designs, identification of the appropriate level for tests and full reporting of outcomes
☒	☐	Estimates of effect sizes (e.g. Cohen's *d*, Pearson's *r*), indicating how they were calculated

Our web collection on statistics for biologists contains articles on many of the points above.

Software and code

Policy information about availability of computer code

Data collection	Flow cytometry data was collected using Bio-Rad ZE5 Cell Analyzer and Everest Software (version 3.1). Absorbency data was collected using the Spectramax iD3 Spectrophotometer (Molecules devices) and Softmax pro software (version 7.0.2).
Data analysis	Flow cytometry data was analyzed using customized Matlab code, which has been deposited on GitHub (https://github.com/AntebiLab/easyflow.git). MATLAB R2020a was used for flow cytometry analysis. Absorbency data was analyzed using Prism 7.0 (Graphpad).

For manuscripts utilizing custom algorithms or software that are central to the research but not yet described in published literature, software must be made available to editors and reviewers. We strongly encourage code deposition in a community repository (e.g. GitHub). See the Nature Research guidelines for submitting code & software for further information.

Data

Policy information about availability of data

All manuscripts must include a data availability statement. This statement should provide the following information, where applicable:

- Accession codes, unique identifiers, or web links for publicly available datasets
- A list of figures that have associated raw data
- A description of any restrictions on data availability

Data Availability:
New plasmids used in this study will be made available for distribution from Addgene (https://www.addgene.org/Xiaojing_Gao/). Annotated plasmid sequences used in this study are provided in the Source Data as GeneBank files. Raw .fcs files are available from the corresponding authors upon reasonable request. Raw experimental data and p-values for each figure are provided as Source Data."

Code availability:
EasyFlow MATLAB code used for flow cytometry analysis is available from the GitHub repository at https://github.com/AntebiLab/easyflow.git.

Field-specific reporting

Please select the one below that is the best fit for your research. If you are not sure, read the appropriate sections before making your selection.

☒ Life sciences ☐ Behavioural & social sciences ☐ Ecological, evolutionary & environmental sciences

For a reference copy of the document with all sections, see nature.com/documents/nr-reporting-summary-flat.pdf

Life sciences study design

All studies must disclose on these points even when the disclosure is negative.

Sample size	All experiments were conducted with at least 3 biological replicates (i.e. N = 3), which is a standard size for such experiments given prior experiments with similar systems (e.g. Gao, X.J, Chong, L.S., Kim, M.S. and Elowitz M.B. Programmable protein circuits in living cells. Science, 361 (1252-1258) DOI: 10.1126/science.aat5062.)
Data exclusions	No data was excluded from the study.
Replication	For each experiment, all data is representative of two independent experiments and replications were successful.
Randomization	Randomization was not relevant in the study since no the same cell line was grown up and divided among different wells prior to transfection.
Blinding	No animal or human participants were used in this study. For cell culture experiments investigators were not blinded. Our experiments extensively use multichannel pipetting for conducting experiments with several conditions at the same time. Hence, it is unlikely that even subconscious bias regarding anticipated results could influence the data. Since many experiments were performed simultaneously it is unlikely that the researcher remembers the identity of any given transfection mix while they conduct the experiment, hence elaborate blinding procedures were deemed unnecessary.

Reporting for specific materials, systems and methods

We require information from authors about some types of materials, experimental systems and methods used in many studies. Here, indicate whether each material, system or method listed is relevant to your study. If you are not sure if a list item applies to your research, read the appropriate section before selecting a response.

Materials & experimental systems

n/a	Involved in the study	
☐	☒	Antibodies
☐	☒	Eukaryotic cell lines
☒	☐	Palaeontology and archaeology
☒	☐	Animals and other organisms
☒	☐	Human research participants
☒	☐	Clinical data
☒	☐	Dual use research of concern

Methods

n/a	Involved in the study	
☒	☐	ChIP-seq
☐	☒	Flow cytometry
☒	☐	MRI-based neuroimaging

Antibodies

Antibodies used	Polyclonal Anti-GFP Dylight 405 antibody (ThermoFischer; catalog# 600-146-215)<br>Polyclonal Anti-Hemagglutinin epitope antibody (Abcam; catalog# ab137838)<br>Donkey polyclonal secondary antibody to Rabbit IgG - H&L conjugated to Alexa Fluor 647 (Abcam, Cat# ab150075)
Validation	From ThermoFischer "GFP Dylight™ 405 Conjugated Antibody was prepared from monospecific antiserum by immunoaffinity chromatography using Green Fluorescent Protein (Aequorea victoria) coupled to agarose beads followed by solid phase adsorption(s) to remove any unwanted reactivities. Assay by immunoelectrophoresis resulted in a single precipitin arc against anti-Goat Serum and purified and partially purified Green Fluorescent Protein (Aequorea victoria). No reaction was observed against Human, Mouse or Rat serum proteins."<br><br>From Abcam " Donkey polyclonal secondary antibody to Rabbit IgG - H&L conjugated to Alexa Fluor 647 was validated by Abcam including relevant controls, routinely running unstained, positive, negative, isotype, viability, Fc-blocking, fluorescence minus one (FMO), and single-staining controls".<br><br>For Polyclonal Anti-Hemagglutinin epitope antibody we validated this antibody against HA, by monitoring the surface staining of cells

transfected with Kir2.1 (containing HA) complexed to RELEASE, with and without the cognate protease. When the cognate protease is not present, Kir2.1 is retained in the ER and we do not observe a surface staining of HA. Subsequently, when protease is present, Kir2.1 is present on the surface and we observe a positive fluorescence signal when cells were incubated with the primary and secondary antibody.

In our laboratory, all flow cytometry experiments include a negative control sample. The negative control is a g0 control plasmid, that is used to ensure that the samples are transfected with the same total mass of plasmid DNA.

Eukaryotic cell lines

Policy information about cell lines

Cell line source(s)	Flp-In™ T-REx™ Human Embryonic Kidney (HEK) 293 cells were purchased from Thermo Scientific (Catlog# R78007)
Authentication	The Flp-In™ T-REx™ Human Embryonic Kidney (HEK) 293 cell line was not authenticated.
Mycoplasma contamination	The cell lines were tested for mycoplasma and were negative.
Commonly misidentified lines (See ICLAC register)	None

Flow Cytometry

Plots

Confirm that:

- [x] The axis labels state the marker and fluorochrome used (e.g. CD4-FITC).
- [x] The axis scales are clearly visible. Include numbers along axes only for bottom left plot of group (a 'group' is an analysis of identical markers).
- [x] All plots are contour plots with outliers or pseudocolor plots.
- [x] A numerical value for number of cells or percentage (with statistics) is provided.

Methodology

Sample preparation	Two days after transient transfection, cells were harvested using FACS buffer (HBSS + 2.5 mg/mL of Bovine Serum Albumin (BSA)). For experiments requiring antibody staining, surface GFP was measured by incubating cells with a 1:1000 dilution of anti-GFP Dylight 405 antibody (ThermoFischer; catalog# 600-146-215) in FACS buffer for one hour at 4°C. For experiments measuring the surface display of Kir2.1, cells were incubated with 1:500 dilution of anti-hemagglutinin antibody (HA, Abcam; catalog# ab137838), followed by incubation with a donkey anti-rabbit IgG conjugated to alexa-647 (Abcam, Cat# ab150075). After staining, cells were washed twice with FACS buffer and then strained using a 40 µm cell strainer. Cells were analyzed by flow cytometry (BioRad ZE5 Cell Analyzer).
Instrument	Flow Cytometry was run on using BioRad ZE5 Cell Analyzer, 4 laser (405/488/561/640 nm) - catalogue# 12004278.
Software	Flow cytometry data was analyzed using customized Matlab code, which has been deposited on GitHub (https://github.com/AntebiLab/easyflow.git).
Cell population abundance	All experiments in this study involve a single cell line. Subpopulation analysis is described below:
Gating strategy	Transfected HEK293 cell population was identified by FSC-A vs. SSC-A gating, and singlets were identified by FSC-A vs. FSC-H gating. For analysis, we selected and compared cells with the highest expression of the co-transfection marker, which was typically mCherry. This was done to have the largest separation between basal reporter autofluorescence from cellular autofluorescence.

- [x] Tick this box to confirm that a figure exemplifying the gating strategy is provided in the Supplementary Information.
