# Supplementary material for: Protease-controlled secretion and display of intercellular signals
Source: Nat Commun. 2022 Feb 17;13:912. doi: 10.1038/s41467-022-28623-y (PMC8854555; doi:10.1038/s41467-022-28623-y)
Supplement: Supplementary file 4 — Description of Additional Supplementary Files [file 41467_2022_28623_MOESM4_ESM.pdf]

Title: Supplementary Data 1:
Description: Experimental details.

Title: Supplementary Data 2:
Description: List of plasmids and the amounts used in this study.
